# ELBOW ULNAR NEUROPATHY: TREATMENT BY ANTERIOR TRANSPOSITION OF THE ULNAR NERVE

**DOI:** 10.1590/1413-785220162404157171

**Published:** 2016

**Authors:** Antonio Tufi Neder, Regina de Azevedo Alves, Arlindo Gomes Pardini, Marcelo Riberto, Milton Mazer

**Affiliations:** 1. Hospital Lifecenter, Belo Horizonte, MG, Brazil.; 2. Hospital Ortopédico, Belo Horizonte, MG, Brazil.; 3. Universidade de São Paulo, Faculdade de Medicina de Ribeirão Preto. Ribeirão Preto, SP, Brazil.

**Keywords:** Ulnar nerve, Cubital tunnel syndrome, Nerve block, Elbow

## Abstract

**Objectives:**

Retrospective clinical evaluation of 31 patients who underwent ulnar nerve decompression at the elbow and subcutaneous anterior transposition*.*

**Methods:**

From January 2000 to December 2013, 71 patients underwent subcutaneous anterior transposition of the ulnar nerve. Thirty-one patients returned for evaluation. The mean follow-up period was 60 months. Patients were evaluated for the degree of satisfaction after surgery, paresthesia, pain, Tinel sign, Froment test and sensitivity test by esthesiometer, muscle strength of the intrinsic muscles and deep flexor of the fifth digit, visual analogic pain scale (VAS) and were subjected to the QuickDash questionnaire*.*

**Results:**

Thirty-nine per cent of patients had compression on the right side and 61% on the left side. Sixty-one percent were idiopathic, 35% post traumatic and 3% had Poems syndrome. Forty-eight per cent of patients were very much satisfied after surgery and 52% were satisfied. Forty-eight per cent had paresthesia after surgery and 52% did not*.*

**Conclusion:**

The ulnar neurolysis of the cubital tunnel with anterior subcutaneous transposition is a safe and effective technique for treating idiopathic or post-traumatic compressive neuropathy, with high success rate and excellent function for activities of daily living. *Level of Evidence IV, Case Series.*

## INTRODUCTION

Compressive syndromes in upper limbs are common diseases in the population, and its high frequency presents challenges to the surgeon in early diagnosis, as in treatment.

The ulnar nerve, in his path through the upper limb, may suffer compression at several levels, but the elbow is the most common site.^1^ ^,^ ^2^ In the elbow, the ulnar nerve compression can happen in five locations: in the Struthers arcade, the medial intermuscular septum, the medial epicondyle, in the cubital tunnel and the aponeurosis of the flexor carpi ulnaris. The most common site of compression is the cubital tunnel.^3^ It is the second most common compression neuropathy of the upper limb.

The ulnar nerve compression can be due to several causes, including chronic compression, fracture sequel or inflammation, tumor, repetitive flexion-extension of the elbow, extended elbow flexion, congenital malformations, systemic diseases such as diabetes, leprosy, etc. In 10-30% of cases, it is idiopathic.

The clinical presentation includes pain, paresthesia and intermittent hypoesthesia in the ulnar nerve territory, with weakness or atrophy of the intrinsic muscles of the hand in more seriouscases.^4^

The diagnosis requires a complete medical history and physical examination. Initially, patients often complain of paresthesia on the little finger and the ulnar side of the ring finger, and as the disease progresses, these symptoms may become more constant and pain can appear. Atrophy of the intrinsic muscles of the hand is a late sign of the disease. Other symptoms are pain in the medial side of the elbow, worsening of symptoms with flexion of the elbow, paresthesia in the back of the hand, reducing the strength of the deep flexors ring and little finger and carpal ulnar flexor.^5^

The cubital tunnel syndrome should be diagnosed with compression of the Guyon's canal, C8 radiculopathy, amyotrophic lateral sclerosis, vascular injuries and peripheral neuropathies secondary to alcoholism.^3^

Elbow X-rays may show fracture sequelae, osteophyte, etc. Electroneuromyography and nerve conduction study may also be useful in the diagnosis and prognosis of the disease.

In the initial phases where there is still high muscle involvement, conservative treatment is the choice, by changing habits that trigger symptoms, anti-inflammatory drugs, orthotics, physiotherapy and antineuritic medication.

Surgery is indicated in cases of failure of conservative treatment, or in patients with severe symptoms or muscle atrophy. There are several different techniques, such as isolated decompression,^5^ decompression with anterior transposition,^6^ ^-^ ^8^ medial epicondylectomy,^9^ ^-^ ^11^ and endoscopic decompression,^12^ ^-^ ^14^ but there is no consensus on which technique is best.^15^

The aim of this study was to evaluate the clinical and functional outcomes of patients undergoing decompression with anterior transposition of the ulnar nerve.

## MATERIALS AND METHODS

We conducted a retrospective study which included patients undergoing subcutaneous anterior transposition of the ulnar nerve at *Hospital Lifecenter* and *Hospital Ortopédico* of Belo Horizonte between 2000 and 2013. The diagnosis of nerve compression of the elbow ulnar nerve was based on a clinical history of paresthesia / hypoesthesia in ulnar nerve territory distribution, weakness or atrophy of the intrinsic muscles of the hand, associated with ulnar nerve conduction velocity through the lower elbow below 50m/s. Exclusion criteria included a follow-up less than 12 months, surgery for cubital tunnel syndrome recurrence, severe neurological or osteoarticular disease of the ipsilateral upper limb, except for other associated compressive neuropathies.

Seventy-one patients met the selection criteria, including 32 men and 39 women. Thirty one patients agreed to participate in the study, two had died, 11 patients were no longer found and the remaining 27 refused to participate for various reasons. Of the 31 patients included in the study, 19 cases (61.29%) had their right side operated and 12 patients had (38.71%) the left side operated. In 18 cases (58.06%) we could not identify a specific etiology, and those were classified as idiopathic, 11 cases were attributed to elbow trauma sequelae and one case was due to tumor compression (lipoma), in a patient with Poems syndrome.

In all patients in this study it was not possible to obtain symptomatic relief with conservative treatment, particularly with activity modification, use of nonsteroidal anti-inflammatory drugs or night immobilization in elbow extension.

The mean follow-up was 60 months (minimum 14, maximum 128 months) and the evaluated patients were asked about the degree of satisfaction postoperatively, being classified as: very satisfied, satisfied, somewhat satisfied or dissatisfied, presence of paresthesia, pain assessment by the visual analog scale for pain^16^ (VAS) and all were subjected to QuickDash score,^17^ in the disorders / symptoms section. Moreover, we assessed the strength of intrinsic muscles and deep flexor of the little finger, compared to the contralateral side, Semmes-Weinstein sensitivity test in the ulnar edge of the hand and the little finger, the Tinel's sign on the elbow and Froment's. Data analysis was performed using SPSS software.^18^

All patients agreed to participate in the study, that was approved by the Hospital Ethics Committee (CAAE) under number 47952115.3.0000.5126.

## RESULTS

The mean follow-up was 60 months, (range 14-128 months). Half of the patients were followed for up to 60 months. Of the patients studied, 15 (48%) considered themselves "very satisfied" and 16 (52%), "satisfied". Paresthesia was present in 15 patients (48%) and absent in 16 (62%).

According to the VAS scale, ranging from zero to 10, 64% of respondents rated their preoperative pain as mild (45% attributed zero value, and 19% assigned value of 2), 12% rated their pain as moderate (values 4 to 7), and 22% rated as severe (with 16% attributed grade 8 and 6% grade 9).

After surgery, 97% of respondents classified their pain as mild (with 55% attributed zero value, 19% attributed value of 1 and 23% assigned value of 2) and 3% rated their pain as moderate, attributing the note 7. (Table 1).

**Table 1:** Assessment of pain by the Visual Analogue Scale.

VAS	Preoperative	Postoperative
0	14 (45%)	17 (55%)
1	0	6 (19%)
2	6 (19%)	7 (23%)
3	0	0
4	0	0
5	1 (3%)	0
6	1 (3%)	0
7	2 (6%)	1 (3%)
8	5 (16%)	0
9	2 ( 6%)	0
10	0	0

VAS: Visual Analogue Scale.

According to EVA, preoperatively, the average was 3.06 (minimum 0, maximum 9) and in the postoperative period it was 0.87 (minimum 0, maximum 7). This difference is statistically significant (Wilcoxon test, p-value = 0.002). With 95% confidence, we can say that the pain score in the postoperative period is significantly lower than preoperatively.

For 52% of the patients, the value of Tinel's sign on the cubital tunnel was negative, and for 48%, it was positive.

As the Froment signal in 27 patients (87%) were negative, and only 4 were positive.

In the postoperative evaluation 9 patients (29%) had muscle strength grade 4 and 22 (71%) showed strength grade 5. The average strength of intrinsic hand muscles and the deep flexor of the little finger was 4.7 (22 patients y showed strength M5 and 9 patients, M4) as compared to the contralateral side.

Regarding sensitivity in the postoperative period, 27 patients (87%) showed sensitivity to the green Semmes-Weinstein monofilament on the ulnar edge of the hand and four patients (13%) showed sensitivity to the blue monofilament. When the assessment was made on the little finger, 26 patients (84%) reported sensitivity to the green monofilament and 5 patients (16%) to the blue monofilament.

The QuickDash^17^ score was applied in all patients, and the average score was 11.8 (0 to 54.5). In this questionnaire, each answered question had a maximum value of 5. These values ​​are transformed into a score of 100, subtracting 1 and multiplying by 25. This transformation is made to compare scores with other scales from 0 to 100. A high score indicates great dysfunction.

Considering quartiles, 75% of patients had Dash scale values ​​between 0.0 and 20.4.

## DISCUSSION

Ulnar nerve compression of the cubital tunnel is the second most common condition within the compression syndromes of the upper limbs, leaving behind only the carpal tunnel syndrome.^1^

Several surgical approaches to this pathology are described in the literature,^2^ ^-^ ^13^ such as neurolysis of the ulnar nerve, subcutaneous transposition, submuscular transposition, intramuscular transposition and partial medial epicondylectomy. However, there is considerable controversy in the literature regarding the best surgical technique.^14^ Most comparative studies have shown similar results and no technique has been shown to be associated with significant differences in the results obtained.^19^

However, Macadam et al.^20^ found a trend towards a better clinical outcome in patients treated with anterior transposition as opposed to simple decompression, requiring prospective studies and additional randomized trials using objective measures to provide statistical support for this finding. Similarly of what has been published in the literature,^6^ ^,^ ^21^ we obtained high rates of success and satisfaction with the subcutaneous transposition technique.

In the present study, we observed a high success rate with this surgical technique, with 48.38% of very satisfied patients and 51.61% of satisfied, while 51.61% have reported some residual paresthesia, but without interfering with activities of daily living. All patients reported improvement in pain as compared to the preoperative period, besides muscle strength M4 and M5, 100% of patients had normal or near normal sensitivity (green and blue Semmes-Weinstein monofilaments). The average QuickDash score was 11.8, the maximum being 54.5, found in one patient who had associated Poems syndrome.

The main limitations of this study were the fact that it is a retrospective study, and we did not have a standardized preoperative evaluation, providing greater rigor to the study. Moreover, many patients refused to do revaluation, culminating in a smaller sample than desired.

## CONCLUSION

Based on the data presented, we conclude that neurolysis of the ulnar in the cubital tunnel with anterior subcutaneous transposition is a safe and effective technique in the treatment of this compressive neuropathy, either idiopathic or posttraumatic, with high success rate in the postoperative and excellent function for activities of daily living.
